# Lipid droplets are versatile organelles involved in plant development and plant response to environmental changes

**DOI:** 10.3389/fpls.2023.1193905

**Published:** 2023-06-23

**Authors:** Imen Bouchnak, Denis Coulon, Vincent Salis, Sabine D’Andréa, Claire Bréhélin

**Affiliations:** ^1^ Centre National de la Recherche Scientifique (CNRS), University of Bordeaux, Laboratoire de Biogenèse Membranaire UMR5200, Villenave d’Ornon, France; ^2^ Université Paris-Saclay, Institut national de recherche pour l'agriculture, l'alimentation et l'environnement (INRAE), AgroParisTech, Institut Jean-Pierre Bourgin (IJPB), Versailles, France

**Keywords:** lipid droplets, environmental stress, triacylglycerol, membrane remodeling, autophagy, lipolysis, heat, post-stress recovery

## Abstract

Since decades plant lipid droplets (LDs) are described as storage organelles accumulated in seeds to provide energy for seedling growth after germination. Indeed, LDs are the site of accumulation for neutral lipids, predominantly triacylglycerols (TAGs), one of the most energy-dense molecules, and sterol esters. Such organelles are present in the whole plant kingdom, from microalgae to perennial trees, and can probably be found in all plant tissues. Several studies over the past decade have revealed that LDs are not merely simple energy storage compartments, but also dynamic structures involved in diverse cellular processes like membrane remodeling, regulation of energy homeostasis and stress responses. In this review, we aim to highlight the functions of LDs in plant development and response to environmental changes. In particular, we tackle the fate and roles of LDs during the plant post-stress recovery phase.

## Introduction

Lipids, and in particular triacylglycerols (TAGs), are the most energetic molecules in cells. Yet their hydrophobic nature requires their storage in a specific intracellular structure, the lipid droplets (LDs). Discovered in the 1880s, LDs have only been widely studied since the 2000s, particularly in mammalian cells because of their involvement in several human diseases such as obesity and atherosclerosis (Coleman, 2020). LDs are composed of a central core of neutral lipids, mainly TAGs and sterol esters, surrounded by a monolayer of polar lipids embedding various proteins. Long considered to be inert fat globules, they are now seen as mobile organelles, found in most of organisms from archaea to eukaryotes (Lundquist et al., 2020).

**Figure 1:**
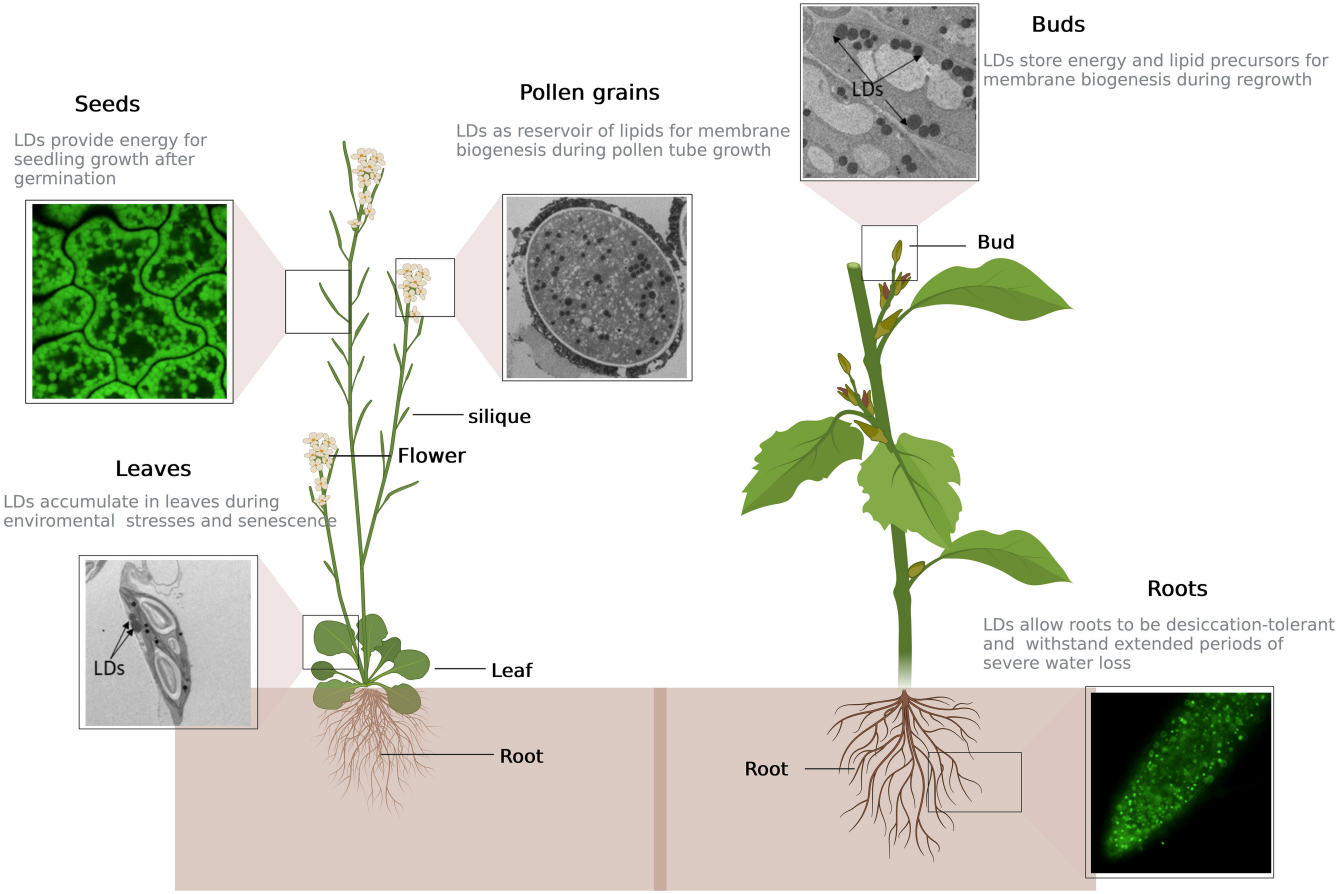
Repartition and function of lipid droplets in different plant tissues. Depending on developmental stage and physiological state of the plant, lipid droplets (LDs) can be present either in seeds or vegetative tissues such as roots, leaves, dormant buds and pollen grains. Top left: cell of Arabidopsis seed stained with BODIPY, a LD-specific dye, and observed by confocal microcopy; center: Arabidopsis pollen grain filled with LDs (in black), observed by electron microscopy; top right: picture of dormant sweet cherry (*Prunus avium*) floral bud by electron microscopy; bottom left: cell of Arabidopsis leaf after five days of nitrogen starvation showing two LDs in contact with chloroplast, observed by electron microscopy; bottom right: LDs in Arabidopsis root tip labelled in green by overexpression of LDAP1-YFP, after two days of nitrogen starvation, observed by confocal microscopy. This figure was created at BioRender.com.

In plants, two compartments are dedicated to the storage of neutral lipids: lipid droplets in the cytosol, also known as oleosomes, spherosomes, or oil bodies, and plastoglobules within plastids (Bréhélin et al., 2007). Cytosolic LDs have been intensively studied in seeds because of their economic importance. However, it is now known that they are also present in pollen and diverse vegetative organs (**Figure 1**). Plastoglobules are thylakoid membrane-bound compartments with a structure similar to LDs. In addition to neutral lipids, they also contain secondary metabolites as carotenoids, plastoquinones, and vitamins (tocopherol and phylloquinone). They are involved in various physiological processes such as lipid metabolism and stress responses (reviewed by van Wijk and Kessler, 2017). The proteome of plastoglobules revealed the presence of FIBRILLINS, a family of structural proteins absent from LDs (Lundquist et al., 2012). The functions of FIBRILLINS and plastoglobules have been recently extensively described (Michel et al., 2021; Arzac et al., 2022; Kim and Kim, 2022) and will not be addressed in this review.

**Figure 2:**
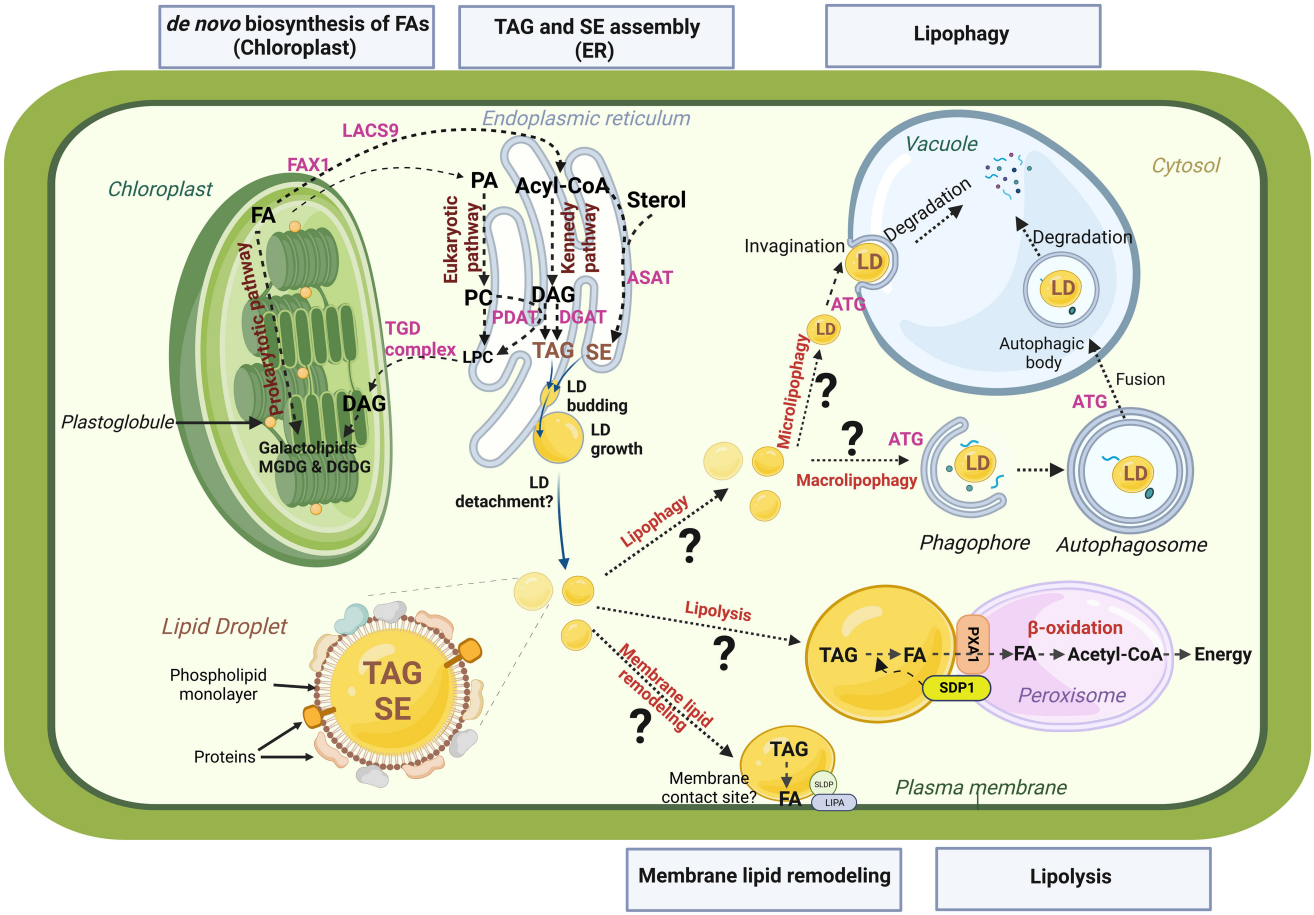
Schematic representation of LD life cycle and potential remobilization pathways. Biosynthesis of fatty acids (FAs) takes place in the chloroplast. Biosynthesis of plastidial galactolipids (MGDG, monogalactosyldiacylglycerol and DGDG, digalactosyldiacylglycerol) occuring within the chloroplast is termed the chloroplastic or ‘‘prokaryotic “ pathway, and the one in the endoplasmic reticulum (ER), that involves phospholipid synthesis (PA, phosphatidic acid; PC, phosphatidylcholine) and subsequent transfer to the chloroplast constitutes the endoplasmic or “ eukaryotic “ pathway. When exported to the cytosol, free FAs are first transported by FA EXPORT 1 protein (FAX1) accross the inner membrane and then converted to activated acyl-CoA by the LONG ACYL-COA SYNTHETASE 9 (LACS9). Acyl-coA are transported to the ER for TAG (triacylglycerol) and SE (sterol ester) assemblies. The major pathway for TAG synthesis is the Kennedy pathway. Diacylglycerol (DAG) is the direct precursor for TAG synthesis. DAG can be converted to TAG by DGAT (DIACYLGLYCEROL ACYLTRANSFERASE) or PDAT (PHOSPHOLIPID DIACYGLYCEROL ACYLTRANSFERASE), using acyl-CoA or PC as acyl donor, respectively. SEs are synthetized from free sterols by the enzymes PSAT (PHOSPHOLIPID STEROL ACYLTRANSFERASE) or ASAT (ACYL-COA STEROL ACYLTRANSFERASE). TAGs and SEs accumulate between the two leaflets of the ER, leading to the formation of a lens-like structure and the budding of a new lipid droplet (LD). Lipolysis and lipophagy are the two main pathways for LD remobilization. Lipolysis involves lipases like SDP1 (SUGAR DEPENDENT PROTEIN 1). PXA1 is a peroxisomal transporter required for uptake of FAs from LD to peroxisome for β-oxidation. Two distinct pathways of lipophagy may be at play: microlipophagy and macrolipophagy. Microphagy involves the invagination of tonoplast to trap LDs within the vacuole. Macrophagy involves the formation of double membrane vesicles called autophagosomes that will sequester LDs and bring them to vacuole for degradation. Both microlipophagy and macrolipophagy involve autophagy related preteins (ATG). TAGs within LDs could also be a source of FAs for lipid membrane remodeling thanks to enzymes that remain to be identified and through organelle contacts maintained by tethering proteins such as SLDP (SEED LD PROTEIN) and LIPA (LD-PLASMA MEMBRANE ADAPTATOR) at LD-plasma membrane connections. This figure was created at BioRender.com.

In recent decades, the mechanistic details of plant LD biogenesis have become clearer (for review see Chapman et al., 2019; Choi et al., 2022; Guzha et al., 2023). Briefly, LD biosynthesis occurs in a three-step process: (i) TAG synthesis at the ER, (ii) TAG accumulation between the two leaflets of the endoplasmic reticulum (ER) membrane leading to the formation of a lens-like structure, and (iii) Budding of the lipid droplet from the ER leaflet into the cytosol. Two pathways permit the synthesis of TAGs: the first one is an acyl-Coenzyme A (acyl-CoA) dependent pathway, called the Kennedy pathway, in which glycerol-3-phosphate is converted to phosphatidic acid (PA) by the sequential esterification of two acyl-CoA to the glycerol 3-phosphate catalysed by two acyltransferases: the GLYCEROL-3-PHOSPHASTE ACYLTRANSFERASE (GPAT) and the *LYSO*-PHOSPHATIDIC ACID ACYLTRANSFERASE (LPAT), and thereafter PA is dephosphorylated by the PHOSPHATIDIC ACID PHOSPHATASE (PAP) to give diacylglycerol (DAG). A finale acylation of DAG to TAG then occurs, catalysed by the DIACYLGLYCEROL ACYLTRANSFERASE (DGAT). The second pathway involves the PHOSPHOLIPID : DIACYLGLYCEROL ACYLTRANSFERASE (PDAT), which does not use acyl-CoA as acyl donor but transfers an acyl group from the phospholipid phosphatidylcholine, to DAG (**Figure 2**; for more details, see Xu and Shanklin, 2016). Neutral lipid accumulation occurs at specific location in the ER, where LDAP-INTERACTING PROTEIN (LDIP) interacts with SEIPIN 2/3 proteins (Coulon et al., 2020; Pyc et al., 2021). During LD budding, LDIP dissociates from SEIPIN complex and interacts with a LD surface protein, LIPID DROPLET ASSOCIATED PROTEIN (LDAP). At the same time, VESICLE-ASSOCIATED MEMBRANE PROTEIN (VAMP) -ASSOCIATED PROTEIN 27-1 (VAP27-1), an ER protein, interacts with SEIPIN2 and/or SEIPIN3 to stabilize the LD-forming complex (Greer et al., 2020). These contact sites between LD and ER membrane have been evidenced by tomography in Arabidopsis leaves (Brocard et al., 2017). It is not clearly determined whether plant LDs may, in a final step, detach from the ER or remain attached to it. The mechanisms that would trigger and control the cleavage of LDs from the ER are totally unknown.

Several proteomics studies conducted on plant LDs from diverse tissues and organ origins, such as hybrid aspen buds (Veerabagu et al., 2021), mesocarp and seed tissues of Chinese tallow (Zhi et al., 2017), nutsedge tubers (Niemeyer et al., 2022), tobacco pollen tubes (Kretzschmar et al., 2018), Arabidopsis seedlings (Kretzschmar et al., 2020), drought-stressed (Doner et al., 2021) or senescent Arabidopis leaves (Brocard et al., 2017) have led to the identification of hundreds of LD-associated proteins. Regularly, scientific reviews describe the newly discovered mechanisms that participate to the regulation of plant LD biogenesis, and list the function of each LD-associated protein. LDs in algae, particularly in the oil-rich diatom *Phaeodactylum tricornutum* or in the model green algae *Chlamydomonas reinhardtii*, are also in the scientist’s spotlight since the two past decades due to their potential use as a platform for biofuel production (reviewed in Kong et al., 2018). Notably, LD proteomes of different algae species have been determined, showing the presence of an abundant structural protein such as MAJOR LIPID DROPLET PROTEIN (MLDP) in *C. reinhardtii* and *Dunaliella salina* (Moellering and Benning, 2010; Nguyen et al., 2011; Davidi et al., 2012) or LIPID DROPLET SURFACE PROTEIN (LDSP) in *Nannochloropsis oceanica* (Vieler et al., 2012), but also of different proteins, more numerous and varied than in land plant LDs, among which are in particular enzymes of the lipid metabolism (reviewed in Goold et al., 2015). While the role of seed LDs as energy reservoirs for the post-germination phase is well known, the function of LDs in non-seed tissues is still far from being elucidated. The aim of the present review is thus to highlight the physiological functions that LDs may play in land plants, from roots to pollen grains (see **Figure 1**), and in particular the role they may have in the plant response to environment changes. Therefore, will we focus our description to LDs of land plants, without any reference to algae LDs.

## LDs are found in many plant organs, where they play various roles

### Seed LDs function in germination and post-germinative growth

#### Seed LD heterogeneity, a sign of functional diversity?

Neutral lipids, mainly TAGs, accumulate to high levels in the seeds of oilseed crops, but are likely present in all seeds. These storage lipids are packaged in micrometer-sized cytosolic LDs distributed in several different types of seed tissue. The abundance and distribution of LDs between seed tissues vary considerably among plant species (Murphy, 2001). In exalbuminous dicotyledons such as rapeseed, sunflower, soybean, and the model plant *Arabidopsis thaliana*, LDs are predominantly stored in the embryo axis and cotyledons. However, the endosperm of these oilseeds, although smaller than embryonic tissues, also contains LDs (Penfield et al., 2004; Miray et al., 2021). In contrast, seeds from albuminous dicotyledons like castor bean and coriander, and from monocots like palm oil or coconut accumulate LDs mainly in their large endosperm tissue, whereas embryonic tissues are relatively poor in lipids. The heterogeneity of seed LDs relates not only to their tissue distribution and abundance but also to their composition. Embryonic tissues and endosperm display contrasting oil composition, as shown by fatty acid (FA) analysis of tissues separated by dissection from various oilseeds such as oil palm and Arabidopsis (Li et al., 2006; Dussert et al., 2013; Miray et al., 2021). A heterogeneous oil composition was also observed within the embryonic tissues by *in situ* mapping of lipid molecular species onto seed sections using matrix-assisted laser/desorption ionization-mass spectrometry imaging (MALDI-MSI). The uneven spatial distribution of TAG species between hypocotyl and cotyledons was indeed demonstrated in various oilseeds (Horn et al., 2012; Sturtevant et al., 2017; Woodfield et al., 2017; Sturtevant et al., 2020). LD heterogeneity is not only related to lipids, but also to proteins associated with the LD surface. While the most abundant proteins of LDs from oilseed species belong to the oleosin (OLE) family (Jolivet et al., 2009; Huang, 2018), two other families of LD-associated proteins, i.e. steroleosins and caleosins, are also characteristic of seed LDs. Oleosins are structural proteins controlling LD size and stability in mature seeds, probably by shielding LD surface and thus preventing LD-LD fusions during seed dessication and rehydration (Siloto et al., 2006; Miquel et al., 2014). On the contrary, caleosins and steroleosins are considered to have enzymatic functions. Steroleosins display hydrosteroid deshydrogenase activity and may play a role in sterol signaling, but their natural substrates remain to be determined (Lin et al., 2002; d’Andréa et al., 2007;Zhang et al., 2016). Caleosins are calcium-binding peroxygenases that associate with seed LDs from many species (Chapman et al., 2012). While the caleosin CLO3 is involved in the production of a phytoalexin in leaves (Shimada et al., 2014, see below), seed-expressed caleosins such as CLO1 modulate LD accumulation and mobilization (Poxleitner et al., 2006; Liu et al., 2022). To date, seed LD proteomes have been deciphered in several species after isolation of LDs from whole seeds (Jolivet et al., 2013; Wang et al., 2016; Kretzschmar et al., 2020). However, when the different seed lipid-rich tissues were separated prior to LD isolation, proteomic analyses revealed tissue specificity in LD protein composition. In jojoba, LDs isolated from the embryonic axis contain a lower amount of oleosins and a higher amount of caleosins and steroleosins than LDs from cotyledons (Sturtevant et al., 2020). In rice, caleosins specifically associate with embryo LDs, in contrast to aleurone layer, which contains only oleosins (Chen et al., 2012). Thus, although the primary function of oilseed LDs is related to energy storage, the coexistence of distinct LD pools in different seed tissues raises the question of whether some LDs with specific distribution and composition may have other functions than providing carbon and energy for seedling establishment. These functions could be related to germinative or post-germinative processes important in both oilseed and non-oilseed species, including provision of lipid building blocks for membrane expansion of organelles such as glyoxysomes (Chapman and Trelease, 1991), oxylipin signaling in response to environmental stress (Hanano et al., 2023), and control of dormancy. This last function, specific to seeds, is supported by a few reports showing that changes in LD structure and composition alter seed dormancy in Arabidopsis. Seed dormancy, an innate state in which viable seeds do not germinate even under favorable environmental conditions, is broken by dry storage or by a cold imbibition. Transgenic lines overexpressing *HSD1* steroleosin exhibit lower cold and light requirements to break seed dormancy than wild type (Li et al., 2007; Baud et al., 2009). More recently, Taurino et al. (2018), showed that *seipin2* mutant seeds display increased dormancy while the final germination rate is not affected. S*eipin2* seeds have enlarged LDs compared to wild-type seeds, due to alteration in LD biogenesis during embryo development, which suggests that LD size may influence the degree of seed dormancy.

#### LD protein remodeling and LD-peroxisome association are central to LD mobilization in seeds

The molecular mechanisms of seed LD mobilization are best described in Arabidopsis and occur during post-germinative growth. Indeed, while the germination is largely driven by the metabolism of non-lipid storage reserves and endosperm storage lipids (Penfield et al., 2004), seedling growth and development relies on the massive and rapid degradation of TAGs that begins once germination is complete, *i.e.* when the radicle has emerged (Eastmond et al., 2000; Graham, 2008). The conversion of seed TAGs to sucrose occurs through sequential metabolic steps catalyzed by well-known enzymes (see Ischebeck et al., 2020; Zienkiewicz and Zienkiewicz, 2020; Choi et al., 2022 for reviews). Briefly, SUGAR-DEPENDENT 1 (SDP1) and SDP1-LIKE (SDP1-L) lipases cleave TAGs to produce DAGs and free FAs (Eastmond, 2006; Kelly et al., 2011). FAs are then degraded by β-oxidation in specialized peroxisomes called glyoxysomes, to generate sucrose through the glyoxylate cycle and the gluconeogenesis pathway. Additional steps are required before peroxisomal β-oxidation, including transport into peroxisomes through the PEROXISOMAL ATP-BINDING CASSETTE TRANSPORTER PXA1 (Zolman et al., 2001) and activation of FA to acyl-CoA by the LONG-CHAIN ACYL-COA SYNTHETASES LACS6 and LACS7 (Fulda et al., 2004). Arabidopsis mutants deficient in SDP1/SDP1-L, LACS6/LACS7 or PXA1 are all defective in seed LD mobilization and require exogenous sucrose for seedling establishment, demonstrating that the primary role of seed LDs is to provide sugars and energy for seedling establishment. SDP1 and SDP1-L account for at least 90% of the TAG lipase activity in Arabidopsis seedlings, but are not involved in the subsequent breakdown of DAGs and monoacylglycerols (MAGs) (Kelly et al., 2011). To date, several candidates have been characterized for lipase activity towards TAG, DAG and MAG, but their contribution to seed oil mobilization has been ruled out or not established (Kim R, et al., 2016; Müller and Ischebeck, 2018; Choi et al., 2022).

The metabolic switch from quiescent to dynamic LDs that occurs during seed germination involves intense remodeling of the LD proteome and the establishment of close associations between LDs and glyoxysomes. The major seed LD proteins, *i.e.* oleosins, caleosins and steroleosins, are progressively degraded and replaced by, among others, LD structural proteins of the LDAP family (Kretzschmar et al., 2020). Remodeling of the LD proteome is most likely a prerequisite for lipolysis, as oleosins are considered to form a protective coat on the surface of LDs, preventing lipases from accessing their TAG substrates prior to seed germination (D’Andrea, 2016). Oleosin degradation during germination depends on their ubiquitination, a post-translational modification also reported for caleosins and steroleosins (Deruyffelaere et al., 2015; Kretzschmar et al., 2020). The addition of a diubiquitin linked on lysine 48 marks oleosins for proteasomal degradation (Deruyffelaere et al., 2015). To be accessible to the cytosolic proteasome for degradation, ubiquitinated oleosins are first dislocated from the LD surface by the CELL DIVISION CYCLE 48A (CDC48A) AAA-type ATPase. Central to this mechanism named LD-Associated Degradation (LDAD) is the adaptor PLANT UBX DOMAIN-CONTAINING PROTEIN 10 (PUX10), which connects CDC48A with ubiquitinated oleosins to promote their extraction from LDs and facilitate their proteasomal degradation (Deruyffelaere et al., 2018; Kretzschmar et al., 2018).

In postgerminative seedlings, LDs are physically and functionally associated with peroxisomes (glyoxysomes). Peroxisomes congregate near LDs to facilitate FA import for glyoxysomal degradation and expansion of glyoxysome membrane (Chapman and Trelease, 1991; Hayashi et al., 2001). Blockage of lipid mobilization in the β-oxidation-deficient *peroxisome defective 1* (*ped1*) mutant or in the lipolysis-deficient *sdp1* mutant results in enlarged peroxisomes, which exhibit membrane invagination at the site of interaction with an LD (Hayashi et al., 2001; Cui et al., 2016). In contrast, exogenous sucrose supplementation reduces the physical interaction between LDs and peroxisomes. These observations suggest that cellular sucrose level regulates the LD-peroxisome association, which in turn controls sugar production from storage lipids (Cui et al., 2016). SDP1 lipase is first localized to the peroxisomal membrane and then delivered to LDs by direct contact or via tubular extensions of the peroxisome membrane, called peroxules (Thazar-Poulot et al., 2015). The retromer trafficking machinery is involved in the formation of peroxules through a mechanism that remains to be identified (Thazar-Poulot et al., 2015). Recently, Huang et al. (2022) reported that FYVE DOMAIN PROTEIN REQUIRED FOR ENDOSOMAL SORTING 1 (FREE1), a component of the endosomal sorting complex for transport (ESCRT), regulates peroxisomal tubulation and SDP1 targeting to LDs. Consistently, *free1* loss-of-function mutant is impaired in LD degradation in postgerminative seedlings. The mechanism is not yet understood, but involves the peroxin PEX11e, a peroxisomal protein that plays key roles in peroxisomal proliferation and tubulation, and SDP1, both of which interact with FREE1.

#### Autophagy is probably also involved in LD mobilization

Apart from lipolysis, LDs can be degraded by lipophagy, which refers to the autophagic degradation of LDs in the vacuole. In plants, lipophagy is poorly described and reported in pollen, and in dark-stressed plantlets (Kurusu et al., 2014; Fan et al., 2019a). Several lines of evidence suggest that lipophagy could also be active during early seedling growth. In Arabidopsis, Avin-Wittenberg et al. (2015) demonstrated that autophagy-deficient *atg5* (*autophagy related gene 5*) and *atg7* seedlings have shorter hypocotyls than wild type when grown in darkness. This phenotype was reversed by providing exogenous sucrose, indicating that autophagy contributes to carbon supply, likely by degrading LDs, in young seedlings. The presence of LDs in the vacuole has been observed by transmission electron microscopy in Arabidopsis and castor bean seedlings (Poxleitner et al., 2006; Han et al., 2020), suggesting that LDs could be degraded in the vacuole. Moreover, ATG8 proteins, which are major players of the autophagy pathway, were shown to interact with seed LD proteins. Marshall et al. (2019) demonstrated that AtOLE1 binds to AtATG8e through an ATG8 interacting motif (AIM). Very recently, in a not yet peer-reviewed manuscript, AtCLO1 was also shown to contain two AIM domains, one of which seems required for its interaction with ATG8b (Miklaszewska et al., 2023). Interestingly, CLO1 deficiency was consistently shown to delay LD mobilization and to prevent the transfer of LDs to the vacuole in post-germinative seedlings. The translocation of LDs into the vacuoles was shown to occur by invagination of the tonoplast, in a process that resembles microautophagy (Poxleitner et al., 2006). Overall, these observations support the hypothesis of an autophagy-dependent vacuolar degradation of seed LDs, but the physiological role and regulatory mechanisms of this alternative pathway to lipolysis remain unknown.

### LDs accumulation in pollen grains is crucial for pollen fertility

Numerous ultrastructural studies have documented the presence of large amounts of LDs within pollen grains (reviewed in Piffanelli et al., 1998). These LDs accumulate during pollen grain maturation (Rotsch et al., 2017) and have been demonstrated to store mostly TAGs (Piffanelli et al., 1997; Hernández et al., 2020). For example, TAGs represent up to 39% of the intracellular lipids of the mature pollen grain of *Brassica napus* (Piffanelli et al., 1997). TAG biosynthesis and LD accumulation seems to be critical for pollen development. Indeed, pollen from mutant impaired in TAG biosynthesis, such as the *dgat1 pdat1* double mutant is sterile (Zhang et al., 2009). The mutation of both transcription factors *wrky2 wrky34* in which genes involved in TAG synthesis are repressed and LD abundancy is reduced in pollen grains (Zheng et al., 2018), also results in pollen sterility (Guan et al., 2014). During pollen grain germination, LDs exit from the grain and accumulate in the growing tube (Rodríguez-García et al., 2003; Zienkiewicz et al., 2013). TAG synthesis during pollen tube growth has been demonstrated by feeding germinating pollens with radioactive ethanol and sucrose, but lipase activity has also been detected in pollen grain and tube (Zienkiewicz et al., 2013). The importance of the TAG lipase activity in pollen germination has been evidenced using the LD-located lipase *obl1* (*oil body lipase 1)* mutant in which the pollen germination is hampered (Müller and Ischebeck, 2018). In addition, *AtSDP1-L* shows exceptionally high expression in mature pollen grains (Kelly et al., 2011), suggesting that AtSDP1-L may be involved in LD breakdown during pollen germination. Indeed, FA composition of TAGs constantly varies during pollen germination, suggesting that TAG mobilization and synthesis both happen in the growing pollen tube (Hernández et al., 2020). In contrast to seeds where TAGs are used as a source of energy for the plantlet post-germinative growth, pollen TAGs rather represent a reservoir of lipids to provide FAs necessary for *de novo* membrane biogenesis during pollen tube growth (Mellema et al., 2002; Ischebeck, 2016; Hernández et al., 2020). The essential role of LDs in pollen fertilization efficiency is exemplified by mutants in LD morphology such as the *seipin2 seipin3* double mutant (Taurino et al., 2018) and the *pald* (*protein associated with lipid droplets*) one (Li et al., 2022), which are impaired in tube germination and pollen longevity, respectively. It can be speculated that FAs are biosynthesized in the pollen grain where nutrients are available, and stored as TAGs within LDs, which are then transported to tip region of the pollen tube where most membrane components are needed. LD transport may happen on the actin filaments present in the pollen tube (Fu, 2015), similarly to what has already been described in root hairs (Veerabagu et al., 2020). As mobile reservoir of FAs, LDs would provide lipids necessary for membrane synthesis during pollen tube growth but also facilitate acyl editing of the lipid membrane and modulate the membrane fluidity in response for example to temperature changes during tube growth (Ischebeck, 2016; Krawczyk et al., 2022a).

### LD abundancy in buds of perennial trees varies during the course of dormancy

Similar to seeds, buds of perennial species establish dormancy, partially desiccate to tolerate frost, and accumulate LDs. Indeed, the presence of LDs in buds has been reported in several perennial species such as birch (Rinne et al., 1999; Rinne et al., 2001), Norway spruce (Guzicka and Wozny, 2003; Guzicka et al., 2018), aspen and poplar (Sagisaka, 1992; Rinne et al., 2011; Veerabagu et al., 2020; Veerabagu et al., 2021), willow (Berggren, 1984), pine (Jordy, 2004) and *Cunninghamia lanceolate* (Xu et al., 2016). LD abundancy is described to vary during winter dormancy progression (Rinne et al., 2001; Sutinen et al., 2009; Sutinen et al., 2012). LDs already present in growing shoot apex, accumulate in quiescent buds during dormancy and are remobilized during bud development once dormancy is released either naturally, or artificially for example by gibberellin (GA_4_) treatment (Rinne et al., 2001; Jordy, 2004; Rinne et al., 2011; Sutinen et al., 2012; Veerabagu et al., 2020; Veerabagu et al., 2021). Recently, Veerabagu et al. (2021) determined the proteome of an LD enriched fraction obtained from poplar dormant buds. Four LDAP isoforms were detected in the bud LD enriched fraction, and the expression of the six *LDAP* genes was highly upregulated in dormant buds compared to developing ones. In addition, LDIP and CLO1 were also present in LD enriched fraction from dormant buds. On the contrary, the expression of three *OLE* genes was high in developing buds but no oleosin was detected in dormant buds. This suggests that LDAPs replace oleosins in LDs during bud maturation, in contrast to what happens during seed development (Kretzschmar et al., 2020). Whether the LD status may regulate bud dormancy, similarly to what has been described in Arabidopsis seeds (Taurino et al., 2018) remains to be investigated.

In addition to their role as energy storage, LDs in buds have been demonstrated to target plasmodesmata during dormancy release. These LDs deliver callose-hydrolyzing 1,3-β-glucanases to remove callose that was deposited during dormancy set to physically close plasmodesmata (Rinne et al., 2001; Rinne et al., 2011). The directional trafficking of LDs to plasmodesmata has been shown in Arabidopsis root hairs to be dependent of the actomyosin system (Veerabagu et al., 2020).

### LDs are also present in roots

LDs can be transiently present in non-dormant vegetative tissues such as leaves or roots, depending on developmental stage and physiological state of the plant (Zienkiewicz and Zienkiewicz, 2020). Yellow nutsedge (*Cyperus esculentus*) is one of the rare plant to accumulate oil in underground tissue (Linssen et al., 1989), and LDs have been shown to accumulate during *C. esculentus* tuber development (Turesson et al., 2010). Comparative proteomic analysis of yellow and purple nutsedge (*Cyperus rotundus*) the closest known relative, whose tubers do not accumulate oil, has revealed that indeed a seed-like proteome is present in yellow but not purple nutsedge (Niemeyer et al., 2022). In fact, the proteome of LD enriched fraction from yellow nutsedge tubers share similarities with the proteome of Arabidopsis seed LDs, notably with the presence of oleosins, steroleosins and SEED LIPID DROPLET PROTEIN (SLDP), that are specifically detected in seed but not leaf LDs (Niemeyer et al., 2022). Because yellow but not purple nutsedge tubers are desiccation-tolerant, it was proposed that oil accumulation probably allows tubers to withstand extended periods of severe water loss. Hensel (1986) has observed the presence of numerous LDs in statocytes of untreated cress roots. In *Arabidopsis thaliana*, it was shown that disruption of *AtSDP1*, the lipase involved in seed TAG hydrolysis, leads to the presence of abundant LDs in roots (Kelly et al., 2013). TAG content in roots of the mutant *sdp1* increases with the age of the plant and can reach more than 1% of dry weight at maturity, a 50-fold increase over the wild type. Such TAG accumulation in *sdp1* roots requires both DGAT1 and PDAT1 and can also be strongly stimulated by the provision of exogenous sugar. However, the physiological role of TAG in *sdp1* roots and of LDs in cress statocytes is unknown.

### LDs in leaves play different functions

#### LD abundancy in leaves depends on the diurnal cycle and the physiological stage of the leaf

As for roots, leaves contain limited amount of TAGs under standard conditions, and LDs were rarely described in leaves until the last decade. Yet, in 2006, Lersten and collaborators (Lersten et al., 2006) provided an overview of relevant literature mentioning the observation of LDs in mesophyll cells as early as 1863. The authors completed this review by searching for the presence of LDs in freehand sections of fresh leaves from more than 300 different species. They observed one or several LDs in leaves of 23% of the surveyed species, evenly distributed within the plant kingdom. Gidda et al. (2016) have shown that the abundancy of LDs in leaves varies during diurnal cycling, with a peak of accumulation at the end of the night. It is thus likely that in most of the species from Lersten’s study, LDs could have been observed at another time of the day, and it is reasonable to suggest that LDs are potentially present in leaves of every plant species, depending on the diurnal cycle and the physiological state of the plant. Leaf LD proteome has a particular composition (Brocard et al., 2017; Fernández-Santos et al., 2020; Doner et al., 2021) compared to the seed LD one. In particular, the main seed LD proteins, oleosins, are absent from leaf LDs, while CLO3 and LDAPs are the two most abundant proteins. The presence of *LDAP* genes in the whole land plant kingdom (de Vries and Ischebeck, 2020) is consistent with the hypothesis that LDs may accumulate in leaves of every land plants. LDs accumulate in senescing leaves (Lersten et al., 2006; Brocard et al., 2017; Zhang et al., 2020; Cohen et al., 2022) where they are supposed to sequester galactolipid-derived FAs before conversion into phloem-mobile sucrose (Kaup et al., 2002). TAG synthesis has been demonstrated to occur in leaves, yet only a very limited TAG content was detected in Arabidopsis senescent leaves (Slocombe et al., 2009; Yang and Ohlrogge, 2009), suggesting this TAG accumulation is transient. It is possible that the abundant LDs observed in senescent leaves have in fact a neutral lipid composition enriched in sterol esters and depleted of TAGs, but this remains to be thoroughly studied.

#### LDs are involved in stomatal aperture and stomatal development

Stomata, pores at the leaf surface that regulate gas exchanges, have been described to contain LDs in their guard cells but also in the subsidiary ones (McLachlan et al., 2016; Pautov et al., 2016; Pautov et al., 2018; Pautov et al., 2022). The disruption of *CLO3* perturbs the opening of Arabidopsis stomata (Aubert et al., 2010), which suggests a role of LDs in the regulation of stomata opening. In fact, LD abundancy correlates with stomatal movements (McLachlan et al., 2016; Pautov et al., 2022) and large LDs in subsidiary cells have been proposed to participate to the turgor pressure and prevent hydropassive stomatal movements (Pautov et al., 2018). McLachlan et al. (2016) have shown that LDs are less abundant in open stomata than in closed ones, and that the regulation of this abundancy is blue light dependent. In the same study, they observed that stomata of mutants impaired in TAG breakdown (*sdp1*, *pxa1* and *cgi- (comparative gene identifier-) 58*) show decreased light-induced opening and conclude that LDs provide the energy necessary for the stomatal opening via the TAG breakdown and the β-oxidation pathway. Stomatal opening is also triggered by heat stress. It has recently been shown that this regulation requires an increase in TAG synthesis and turnover (Korte et al., 2023), suggesting that LD dynamics are important for efficient stomatal opening not only at dawn, but also in response to heat stress. Measurement of LD abundancy in guard cells in response to heat will be needed to confirm this hypothesis.

In addition to playing a role in the regulation of the stomatal aperture, LDs are also involved in the stomatal development. Ge et al. (2022) observed that LDs are abundant in the meristemoid cell that is at the origin of the stomata, and that their abundancy decreases with stomatal maturation. They have shown that LD dynamics is critical for the stomata development. RABC1, a RAB GTPase involved in stomata morphogenesis that locates at the ER, is targeted to LDs when both LD biogenesis is induced and RABC1 activated by a GUANINE NUCLEOTIDE EXCHANGE FACTOR protein (GEF). The inactivation of RABC1, by the mutation of either *RABC1* or *RABC1GEF1* genes leads to a defect in stomatal morphology and function, linked with the presence of aberrant large LDs in guard cells. RABC1 regulates the localization of SEIPIN2/3 to LDs, and the *seipin2 seipin3* double mutant also display large LDs in deformed guard cells. It is proposed that LDs provide neutral lipids, which mobilization is necessary for guard cell formation (Ge et al., 2022).

### LDs represent specialized storing compartments for non-TAG neutral lipids

In latex producing plants, such as rubber trees (*Hevea brasiliensis*) and rubber dandelion (*Taraxacum brevicorniculatum*), latex accumulates in rubber particles, organelles with a structure similar to LDs. The hydrophobic core of rubber particles is mainly composed of poly(*cis*-1,4-isoprene) delimited by a monolayer of phospholipids (Cornish, 2001) associated with at least two major proteins, the small rubber particle proteins and the rubber elongation factor (Dennis and Light, 1989; Oh et al., 1999). These two proteins being homologs to the LDAPs (Gidda et al., 2013; Berthelot et al., 2014), it seems reasonable to postulate that rubber particles are LDs specialized in the storage of latex. Yet, it is still unclear whether the formation of rubber particles occurs at the ER, similarly to the biogenesis of TAG-containing LDs (Dai et al., 2013; Guzha et al., 2023).

Jojoba (*Simmondsia chinensis*) is a peculiar plant that stores up to 60% of seed weight of wax esters instead of TAGs (Miwa, 1971). These wax esters, composed of a long-chain fatty acid esterified to a long-chain fatty alcohol, accumulate in cotyledons in what was first called wax bodies (Rost and Paterson, 1978). There are hydrolyzed after seed germination to provide energy through the sequential action of a wax ester hydrolase, a fatty alcohol oxidase and a fatty aldehyde dehydrogenase to convert fatty alcohols to fatty acids before β-oxidation (Rajangam et al., 2013). In addition to oleosin and caleosin proteins, which are classically found in seed LDs, LDAP1 has also been identified in LDs from Jojoba seeds (Sturtevant et al., 2020). This suggests a peculiar role of LDAP1 in ensuring correct packaging of particular hydrophobic compounds such as wax esters in Jojoba seeds or isoprenoids in rubber plants because LDAP proteins are usually absent from TAG accumulating seeds.

The neutral lipid core of LDs provides favorable storage site for fat-soluble vitamins. Vitamin E and vitamin A accumulate in LDs from animal cells (Welte and Gould, 2017). In plants, vitamin E and carotenoids (the precursors of vitamin A) are described to accumulate in plastoglobules inside plastids rather than in cytosolic LDs (Deruere et al., 1994; Vidi et al., 2006; Zita et al., 2022). Some studies reported the presence of tocopherols in seed LDs (Zaaboul et al., 2018; Lopez et al., 2023). It remains to be determined with precision whether plant cytosolic LDs contain other vitamins and which ones. Additional non-polar compounds probably accumulate within different peculiar plant species LDs, such as, for example, triterpenoid esters in LDs from tea leaves (Zhou et al., 2019).

### LDs participate to the plant response to abiotic and biotic environmental stresses

The implication of LDs in stomata aperture is an illustration of the role played by LDs in plant response to stress. In fact, LDs have been proposed to represent a general mechanism developed not only by land plants, but shared across the green lineage, for stress resilience (de Vries and Ischebeck, 2020). In the following paragraphs, we aim at illustrating such involvement of LDs in plant response to stress.

### LDs accumulate in response to some abiotic stresses: the example of thermal stresses

The fluidity of the lipid bilayer of cell membranes is altered by temperature. In response to diurnal and seasonal temperature fluctuations, plants compensate for membrane fluidity imbalance by decreasing or increasing membrane lipid unsaturation degree in response to high or low temperature stress, respectively. Additional membrane alterations occur under changing temperature conditions. Heat stress induces the production of reactive oxygen species, which promote membrane lipid peroxidation (Farmer and Mueller, 2013; Pospisil, 2016). Freezing stress generates intracellular ice, which results in severe dehydration and membrane deformations causing leakiness and loss of bilayer structure (Uemura et al., 1995). Temperature stresses induce transcriptional and metabolic changes important for long-term plant acclimation (Fowler and Thomashow, 2002; Falcone et al., 2004), but also rapid remodeling of membrane lipids important for basal tolerance (Burgos et al., 2011; Li et al., 2015). However, lipid remodeling does not apply only to membrane lipids but also to reserve lipids, which are consistently accumulated during heat and freezing stresses.

In Arabidopsis, Moellering et al. (2010) showed that freezing induces a 7.5-fold increase of leaf TAGs. They demonstrated that this TAG accumulation depends on the galactolipid remodeling of chloroplast membranes by the galactolipid:galactolipid galactosyltransferase SFR2. SFR2 stands for SENSITIVE TO FREEZING 2, and as this name clearly indicates, Arabidopsis plants carrying mutations in *SFR2* are not able to resist freezing and show severe intracellular damage with rupture of chloroplast membranes (Fourrier et al., 2008). SFR2 is localized to the outer chloroplast envelope and catalyzes the conversion of MGDG to di-, tri-, and tetragalactosyldiacylglycerol (DGDG, TGDG, and TeDG, respectively) by transglycosylation, resulting in the concomitant production of DAG. SFR2 deficiency impairs freezing-induced accumulation of TAGs, notably those containing the fatty acid C16:3, which is predominately esterified to MGDG in Arabidopsis (Moellering et al., 2010). This result indicates that DAG produced by SFR2 from MGDG is further acylated to TAG. The enzyme catalyzing the conversion of DAG to TAG upon cold exposure has been identified as DGAT1 in both Arabidopsis and *Boechera stricta* (Arisz et al., 2018; Tan et al., 2018). Overexpression of AtDGAT1 in Arabidopsis enhances freezing tolerance of seedlings (Arisz et al., 2018). Conversely, Arabidopsis *dgat1*-deficient mutants exhibit reduced tolerance to chilling (3 weeks at 4°C) or freezing (40 min at -8°C) stresses than the wild type (Tan et al., 2018), demonstrating that the conversion of DAG to TAG by DGAT1 is critical for plant freezing tolerance. Together, SFR2 and DGAT1 are believed to contribute to membrane stabilization during freezing stress by decreasing MGDG, DAG, and PA content. Indeed, these lipids tend to form a nonlamellar HII-type phase promoting the shrinkage of membrane structure and membrane ionic leakage (Du and Benning, 2016). TAGs that are consequently produced from MGDG-derived DAGs are most likely stored in cytoplasmic LDs. Indeed, Gidda et al. (2016) showed that the abundance of LDs increases nearly 10-fold in Arabidopsis leaves after 24h of exposure to 4°C. Transcriptional analyses of Arabidopsis *LDAP1*, *LDAP2*, and *LDAP3* in response to cold stress suggest that cold-induced LDs are decorated by LDAP1 and LDAP3 but not by LDAP2. However, LD accumulation induced by cold stress is reduced in *ldap3* but not *ldap1* mutant, suggesting a specific role of LDAP3 in cold-induced LD proliferation (Gidda et al., 2016). Another LD-associated protein, EARLY RESPONSE TO DEHYDRATION ERD7, has been identified for its role in cold stress response (Doner et al., 2021). Arabidopsis lines deficient in ERD7 and its homologs are more susceptible to freezing than the wild type, perhaps due to decreased membrane flexibility (Barajas-Lopez et al., 2021). ERD7 does not regulate LD abundance and morphology under normal growth conditions (Doner et al., 2021). Further investigation will be required to determine the exact role of ERD7, especially during stress conditions.

Heat stress also leads to TAG accumulation in plant tissues, as has been consistently reported in different species (Higashi and Saito, 2019). In Arabidopsis leaves exposed to heat stress at 37°C, TAGs accumulate rapidly in the first 3 h and reach a steady state level after 6 h (Mueller et al., 2015; Yurchenko et al., 2018). Compared to other abiotic stresses such as cold, salt, drought, and high light, heat is the strongest inducer of TAG accumulation after short-term stress exposure (Mueller et al., 2015). While the level of total fatty acids remains unchanged during heat stress in Arabidopsis, indicating that heat-induced TAG accumulation is not driven by massive *de novo* fatty acid synthesis (Mueller et al., 2015), lipidomic studies revealed significant lipid remodeling. In Arabidopsis leaves and seedlings, a temperature shift from 22°C to 37°C or 45°C for 2 h to 1 day leads to the accumulation of TAG species enriched in polyunsaturated fatty acids (PUFAs), such as TAG 54:7, TAG 54:8, and TAG 54:9 (Higashi et al., 2015; Mueller et al., 2017; Shiva et al., 2020). Similar observations were also reported in wheat and Begonia leaves, and castor bean seedlings (Narayanan et al., 2016; Sun et al., 2022; Zhang et al., 2022). A 2-fold accumulation of TAGs in response to 2-h exposure to 37°C was also reported in *Nicotiana tabacum* germinating pollen, but heat-induced TAGs in pollen, unlike those in leaves, were enriched in saturated FAs (Krawczyk et al., 2022a). In leaves, the increase of PUFAs in TAGs is paralleled by their decrease in chloroplastic membrane lipids such as MGDG, sulfoquinovosyldiacylglycerol, and phosphatidylglycerol (Higashi et al., 2015). This observation suggests that heat-induced TAGs are mainly derived from chloroplastic 16:3 and 18:3 PUFAs. A nice demonstration of this premise was provided by Mueller et al. (2017) by comparing PUFA levels in TAGs in Arabidopsis *fad* (*fatty acid desaturase*) *3* and *fad7/8* lines, which are deficient in ER- and plastid-located ω-3 desaturases, respectively. Indeed, heat-induced accumulation of 16:3 and 18:3 PUFA in neutral lipids depends on FAD7/8 desaturases, which produce these trienoic PUFAs in chloroplasts, but not on FAD3 desaturase, which synthetizes them in the ER. Consistently, Higashi et al. (2018) showed that a lipase named HIL1 (HEAT INDUCIBLE LIPASE 1), which catabolizes MGDG to produce 18:3 free FA, is involved in the heat-induced remodeling of MGDG and DGDG, and the concomitant accumulation of PUFA-containing TAGs. The reduction of the heat-induced galactolipid turnover in the *hil1* mutant is associated with a higher sensitivity to heat stress, compared to the wild type. They also established that *HIL1* expression is induced by heat, and that HIL1 localizes to the chloroplast (Higashi et al., 2018). Thus, the first committed step of chloroplastic lipid remodeling induced by heat stress is mainly catalyzed by HIL1, without excluding a minor contribution of other enzymes such as SFR2, as suggested by Mueller et al. (2017). Ultimately, FAs released from chloroplastic membranes are incorporated into cytosolic TAGs and accumulated in LDs. The PHOSPHOLIPID : DIACYLGLYCEROL ACYLTRANSFERASE PDAT1 is required for TAG accumulation in response to heat stress, but DGAT1, the PHOSPHATIDYLCHOLINE : DIACYLGLYCEROL CHOLINE-PHOSPHOTRANSFERASE PDCT, and PHOSPHATIDIC ACID PHOSPHOHYDROLASES PAH1/PAH2 are not (Mueller et al., 2017). PDAT1 is also involved in heat-induced TAG accumulation in guard cells (Korte et al., 2023). Thus, it appears that PUFAs released from chloroplast membranes are mainly channeled into phosphatidylcholine before being sequestered into TAGs. Interestingly, *pdat1* seedlings are more sensitive to heat stress, indicating that PDAT1-mediated TAG accumulation is important for plant tolerance to high temperature (Mueller et al., 2017). Heat-induced TAGs accumulate predominantly in cytosolic LDs in Arabidopsis and castor bean (Mueller et al., 2015; Zhang et al., 2022). Consistently, Gidda et al. (2016) reported massive proliferation of LDs after 1-h exposure at 37°C. They also showed that LDAP1 expression is induced in response to heat, and LD proliferation in *ldap1* mutant is reduced, suggesting a role of LDAP1 in the LD dynamics induced by heat (Gidda et al., 2016).

Plants accumulate TAGs and LDs in response to other various abiotic stresses such as salinity, drought, high-light, starvation, etc., but the role of neutral lipids in general, and of LDs in particular, is far less documented in these stresses. Plants share common responses against drought and salt stresses since they both induce a severe dehydration (for review Krasensky and Jonak, 2012). A 2.5-fold increase of TAG has been reported when 2 week old Arabidopsis seedlings were submitted either to a 2h drought or 125 mM NaCl treatment for 2h (Mueller et al., 2015). This increase in TAG content is paralleled by a significant increase of the LD number in leaves (Doner et al., 2021). Similarly, an increase of LD abundancy was observed in mesophyll cells of the rocket *Eruca sativa* after a saline stress (Corti et al., 2023). Several proteins associated with lipid synthesis have been identified in the LD proteome of microalgae *Parachlorella kessleri* submitted to a salt stress (You et al., 2019). Specifically, an acetyl-CoA carboxylase, LPAT, 3-ketoacyl-CoA synthase, LACS and diacylglycerol kinase were identified. These findings suggest that LDs play a role in the synthesis of phospholipids and membrane remodelling in response to salt stress, at least in algae. A proteomic study (Doner et al., 2021) performed on LDs purified from drought-stressed Arabidopsis leaves, showed an enrichment of LD proteins that have been previously associated to the plant response to stress, such as CLO3, LDAP1, LDAP3, DOX1 and ERD7. CLO3, also named RESPONSIVE TO DESICCATION 20 (RD20), has previously been shown to play key function in the plant response to diverse stresses (Blée et al., 2014). Notably the knock out mutant *rd20* is less resistant to drought stress probably because of its alteration of stomatal aperture (Aubert et al., 2010). Similar to *CLO3*, *LDAP* gene expression is induced by drought stress (Aubert et al., 2010; Kim E, et al., 2016). Arabidopsis *ldap1* and *ldap3* mutants are less tolerant to drought stress while the overexpression of some of the Arabidopsis or *Taraxacum brevicorniculatum LDAP/SRPP* genes confers higher resistance to Arabidopsis plants (Kim E, et al., 2016; Laibach et al., 2018). It is not clear whether the impact of LDAP expression on the plant drought tolerance is due to a direct role of the LDAP proteins in induction of drought tolerance or is a consequence of their involvement in the LD biogenesis and LD size regulation.

### LDs in the context of plant-pathogen interactions: friend or foe?

Examples of an involvement of LDs in plant response to pathogens are really scarce, but the expression of several LD-related genes such as *LDAP1, CLO3* or *α-DOX1* is upregulated in Arabidopsis leaves infected by the fungi *Botritys cinerea* (Sham et al., 2014), which suggests that LD biosynthesis is induced either by the fungi for hijacking of the host lipid metabolism, or by the plant as a defense against the infection. Indirect evidences of LD accumulation in response to pathogens have also been obtained by following the content of neutral lipids, which are stored mainly within LDs. Recently, an important increase of TAG content (more than 6 fold higher compared to the control plant) was observed in Arabidopsis roots inoculated with the fungi *Verticillium longisporum* (Schieferle et al., 2021), and also in distal leaves of the same infected plants despite the absence of the fungi in these leaves. This suggests the existence of a systemic signal inducing TAG synthesis in leaves. The bacteria *Pseudomonas syringae* also induces an increase of TAG content in infected leaves (Zoeller et al., 2012; Schieferle et al., 2021). Interestingly, while the FA composition of TAGs synthesized in response to *P. syringae* infection clearly suggests that the TAGs derive from the membrane degradation locally provoked by the pathogen, similarly to the process generally described upon abiotic stress, the composition of TAGs accumulating in leaves in response to *V. longisporum* is peculiar, with fewer insaturations. This could result from the involvement of an alternative TAG synthesis pathway, but this remains to be elucidated. Indeed, the study of *dgat1* and *pdat1* mutants suggests that TAGs induced by *V. longisporum* are produced independently of these two acyl-transferases. In animal cells, LDs have been demonstrated to be hijacked by some bacteria, for example *Mycobacterium* or *Chlamydia*, to serve as major source of energy and carbon (reviewed in Roingeard and Melo, 2017). But LDs are also described as forming the “first-line of intracellular defense” against some bacteria in mammalian cells by establishing contacts with bacteria and providing antimicrobial peptides (Bosch et al., 2020). Whether the TAG accumulation in Arabidopsis roots and leaves in response to *V. longisporum* is induced by the fungi to divert the cell metabolism, or by the plant cell as a defense response still needs to be investigated.

Hara-Nishimura and colleagues have demonstrated the involvement of LDs in the production of antifungal molecules such as oxylipins (Shimada et al., 2014). The authors first determined that LDs accumulating in leaves upon infection by the pathogenic fungus *Colletotrichum higginsianum* contain CLO3 and the α-DIOXYGENASE α-DOX1. Then, CLO3 and α-DOX1 were shown to interact and to convert C18:3 fatty acid into oxylipin 2-hydroxy-octadecatrienoic acid, which has antifungal properties. Later, PAD3, an enzyme involved in the synthesis of the antimicrobial phytoalexin camalexin was also demonstrated to relocate to LDs upon infection by *P. syringae* (Fernández-Santos et al., 2020). Taken together, these results suggest that LDs can play a role in plant defense as “subcellular factories” producing antimicrobial compounds, at least in Arabidopsis leaves.

The work of Coca and San Segundo (2010) on the CALCIUM DEPENDENT PROTEIN KINASE 1 (CPK1) also suggested an involvement of LDs in the plant response to biotic stress. Arabidopsis *cpk1* knock out mutants are indeed more sensible to fungi while CPK1 overexpressors are more resistant. The localization of CPK1 in LDs in addition to peroxisomes, thus suggests a role of LDs in resistance to pathogen, albeit the function of the association of CPK1 with LDs is still obscure.

In contrast, Yang et al. (2021) have shown that the oomycete *Phytophtora infestans* induces the degradation of LDs in guard cells in order to induce and maintain stomatal opening of potato leaves, thus facilitating the emergence of sporangiophores from opened stomata.

As obligatory parasites, viruses have developed tactics to reroute host cellular functions for their own benefits. In particular, animal (+) RNA viruses harness lipids and manipulate cell membranes to create membranous compartments called viral replication compartments (VRCs) (Zhang et al., 2019). In particular, Laufman et al. (2019) have nicely described the LD hijacking by poliovirus: poliovirus recruits LDs to the forming viral organelles to enable transfer of FAs originating from the lipolysis of LD-stored triacylglycerol to VRCs, where they are incorporated as polar lipids. LD recruitment was shown to be driven by viral proteins that tether the VRCs to the LDs while other viral proteins interact with the host lipolysis machinery to channel FAs into the phospholipids that constitute the VRC membranes. The inhibition of the contact sites formation between LDs and VRCs impedes poliovirus replication (Laufman et al., 2019). Similarly to animal ones, plant RNA viruses have the capacity to induce the proliferation of host endomembranes derived from ER, chloroplasts, mitochondria, peroxisomes, or vacuole, for VRC formation (Jin et al., 2018; Medina-Puche, 2019). Interestingly *Tomato bushy stunt virus* (TBSV), which is one of the rare plant (+) RNA viruses that can multiply in the model host yeast (Nagy et al., 2016), was shown to replicate faster in the yeast *pah1* mutant strain in which the *PHOSPHATIDATE PHOSPHATASE PAH1* gene is deleted (Chuang et al., 2014). In addition, TBSV accumulation was hindered in *N. benthamiana* plants overexpressing the Arabidopsis *PAH2* gene. PAH enzymes play key roles in cellular decisions about membrane biogenesis versus lipid storage, thus these results emphasize the importance of the host lipid metabolism regulation by some plant viruses for their replication. Yet to our knowledge, no data are available demonstrating any links between LDs and plant virus infection, and the possible involvement of LDs in plant virus infection, either at the benefit of the virus or as a plant defense mechanism against the virus, remains to be explored.

### Diverse pathways participate to LD biogenesis in response to environmental stress

As mentioned above, during environmental stresses like nutrient starvation, or heat, LDs accumulate in cells to later disappear upon favorable environmental conditions. Besides the peculiar role of LDs as reservoir for the oxylipins defense compounds (Shimada et al., 2014), the origin and the exact function of this LD transient formation upon stress is still unclear. The process leading to TAG accumulation seems to differ depending on the stress type, since heat, drought and salt stress induce a rapid TAG accumulation, while TAG synthesis is induced only after several days of exposure to cold, high light or osmotic stress (Mueller et al., 2015). Under oxidative stress conditions, reactive oxygen species are produced, inducing loss of the membrane bilayer integrity, deterioration of the organelles, and leading to generation of cytotoxic free FAs. These FAs released from the membranes are then packed as TAGs and sterol esters in LDs to prevent free FA-induced toxicity (Fan et al., 2013; Xu and Shanklin, 2016). In Arabidopsis plants grown under standard conditions, the disruption of PDAT1 in the *tgd1 (trigalactosyldiacylglycerol 1)* mutant causes severe decreases in TAG levels with concomitant increases of the content of free FAs, DAG and membrane phospholipids, and premature cell death in growing leaves and floral organs (Fan et al., 2013). This demonstrates the crucial role of TAG biosynthesis and storage for lipid homeostasis and free FA detoxification. Mueller et al. (2017) have shown that PDAT1 but neither DGAT1, nor PDCT, and nor PAH1/PAH2, is required for TAG accumulation in response to heat stress. This confirms that TAGs accumulated under heat stress do not originate from the Kennedy pathway but rather derived from lipid membrane remodeling and FA recycling. Indeed, under stress conditions, the concentration of toxic free FAs is highly regulated by feedback inhibition of FA biosynthesis by oleic acid-ACYL CARRIER PROTEIN (18:1-ACP), which inhibits plastidic ACETYL-COA CARBOXYLASE (Flügge et al., 2011). In contrast to heat stress, upon freezing, DGAT1 is an essential player of TAG formation (Arisz et al., 2018). During cold acclimation phase, TAG accumulates in *B. stricta* seedlings from conversion either of phosphatidylcholine or of MGDG, depending on the ecotypes, while some TAGs are formed with newly synthesized FAs (Arisz et al., 2018).

### LD degradation probably participate to plant stress recovery upon return to normal environmental conditions

Plant tolerance to stress depends not only on the efficient elimination of damaged organelles/membranes, but also on the capacity to remodel functional compartments upon return to normal conditions. Recent studies have revealed immediate rapid disappearance of LDs post-stress (Mueller et al., 2015; Graef, 2018; Zienkiewicz and Zienkiewicz, 2020). Thus, it has been postulated that LD degradation is involved in stress recovery upon return to normal environmental conditions. The essential role of LDs for the resumption of cell growth is documented in yeast and microalgae (Graef, 2018; Lee et al., 2020; Zienkiewicz and Zienkiewicz, 2020), and the mobilization of seed LDs during post-germinative growth is well described (Kelly et al., 2011; Deruyffelaere et al., 2015; Thazar-Poulot et al., 2015; D’Andrea, 2016; Deruyffelaere et al., 2018). However, the mechanisms involved in the plant recovery phase after a period of stress, and leading to a rapid built of new organelle membranes from damaged ones, are still an enigma. In particular, it is unclear whether FAs transiently stored as TAGs in LDs are hydrolyzed during stress recovery for energy supply or recycled as source of FAs for lipid synthesis to restore the integrity of membranes. Probably both processes happen.

Under deprivation of nutrients, which induces growth arrest, LD accumulation allows the storage of the excess carbon produced by photosynthesis, and will provide energy and carbon to quickly restart when the conditions get better. Fan et al. (2019b) have suggested that under starvation conditions, the degradation of membrane lipids and the subsequent formation of TAGs in leaves may participate to the maintenance of energy homeostasis.

Very little is known about the degradation of plant LDs after stress and the lipid metabolism associated to post-stress recovery. Mueller et al. (2015) reported that TAG mobilization occurs in less than one day upon recovery from heat stress suggesting a rapid and acute degradation of stress-induced TAGs during the recovery phase. Lipids stored in LDs are degraded by two distinct pathways: lipolysis which relies on the action of lipases, and lipophagy, a specialized form of autophagy (**Figure 2**).

Lipolysis, coupled to β-oxidation, is the main pathway that supply energy and carbon for seedling growth after germination (Kelly et al., 2011; Thazar-Poulot et al., 2015). So far, the best characterized TAG lipases directly involved in LD mobilization in plants are SDP1 and SDP1-L proteins (Eastmond, 2006; Quettier and Eastmond, 2009; Kelly et al., 2011). Previous studies have shown that the expression level of *AtSDP1* increases during natural leaf senescence (Troncoso-Ponce et al., 2013) and that disruption of *AtSDP1* leads to TAG accumulation also in vegetative tissues, such as leaves, stems, and roots (Kelly et al., 2013; Fan et al., 2014). Later, SDP1-dependent lipolysis was proposed to participate in leaf TAG mobilization from dark-induced LDs (Fan, 2017). *AtCGI-58* transcript levels are up-regulated during leaf senescence (Troncoso-Ponce et al., 2013), and *atcgi-58* mutant shows a significant increase in TAG content of leaf mesophyll cells (James et al., 2010). Similarly to mammalian CGI-58 that has been shown to regulate TAG breakdown by activating ATGL (Lass et al., 2006), Arabidopsis CGI-58 has been suggested to promote TAG turn-over by activating PXA1 hydrolysis activity (Park et al., 2013). Many genes putatively coding for lipases are expressed during leaf senescence (Troncoso-Ponce et al., 2013), suggesting that more lipases could be involved in LD turnover during leaf senescence or in response to environmental stress, yet their activity remains to be described.

Post-germinative LD degradation is delayed in LDAP1-deficient Arabidopsis plants (Gidda et al., 2016), suggesting that LDAP1 (and possibly other LDAPs) might orchestrate the LD docking and activation of actors of LD/TAG degradation, similar to the function of PERILIPINs (PLINs) in mammalian cells (D’Andrea, 2016). Moreover, LDAPs are differentially up-regulated by diverse stresses and are associated with a better tolerance to drought (Gidda et al., 2013; Kim et al., 2016a). On the basis of these observations, we anticipate that LDAPs might be involved in LD remobilization and plant fitness upon stress recovery not only via their structural function on LDs, but also by promoting the formation and/or recruitment of protein modules dedicated to this mobilization.

In addition to lipolysis, lipophagy is the alternative LD degradation pathway during which LDs are specifically targeted by the autophagy machinery, as reported in mammals, yeast and algae (Singh et al., 2009; Zhao et al., 2014; Graef, 2018). Two distinct types of autophagy have been described in plants: macroautophagy and microautophagy (**Figure 2**). Macroautophagy involves the formation in the cytosol of double membrane structures called autophagosomes (Yoshimoto and Ohsumi, 2018). During their formation, autophagosomes entrap different cargo and carry them to the lytic vacuole lumen where they will be released after fusion of the autophagosome outer membrane with the tonoplast. Microautophagy is characterized by the invagination of the tonoplast to trap cytoplasmic material and create autophagic bodies within the vacuole lumen (Masclaux-Daubresse et al., 2020). Diverse ATG proteins participate in the induction of macro- and micro-autophagy. Plant autophagy is highly induced by various abiotic stresses, including nutrient deprivation (Janse van Rensburg et al., 2019), dark-induced starvation (Fan et al., 2019b), drought (Bao et al., 2020), and biotic stresses as pathogen infection (Dagdas et al., 2016). Several studies have provided evidence of an important role of autophagy in LDs remobilization in non-seed tissues (Kurusu et al., 2014; Fan et al., 2019a). Potential role of lipophagy in pollen grain maturation was described in rice (Kurusu et al., 2014), where *Osatg7* mutant defective in autophagy showed lower contents of TAGs and LDs in mature pollen, but a higher accumulation of LDs in tapetal cells compared to wild type plants. These results, suggest that ATG7 may be responsible for LD degradation and TAG remobilization in the tapetum. More recently, Zhao et al. (2020) showed that silencing of *ATG2* and *ATG5* leads to a lower number of autophagosomes at the germinative pollen aperture and is accompanied by inhibition of pollen germination and significant accumulation of TAGs and DAGs in pollen grains. Autophagy is also implicated in LD breakdown in senescent leaves of watermelon (*Cirullus lanatus*) (Zhang et al., 2020) and in LD turnover in Arabidopsis leaves during dark-induced starvation (Fan et al., 2019a). Indeed, ultrastructural analysis performed on dark-treated *atsdp1-4* leaves, defective in cytosolic lipolysis, showed accumulation of LDs in the central vacuole, probably resulting from the trapping of LDs by invagination of the tonoplast during microlipophagy (Fan et al., 2019a). In addition, macro-autophagy has been shown to be involved in the degradation of lipids originating from the endomembranes of various organelles, except for those of the chloroplasts (Fan et al., 2019a; Havé et al., 2019). Whether lipophagy occurs in response to other stresses and contribute to plant recovery remains an open question, which requires further investigations.

## Conclusion

While described in non-seed tissues since more than a century, the physiological roles of LDs in vegetative cells have been under investigation since only few years. Their presence in almost every organs and tissue of the plant suggest a wider function than their primary role as reservoir of energy for post-germinative growth. In fact, plant LDs must be considered as dynamic versatile organelles with numerous roles. Of course, they principally play functions related to lipid metabolism such as protection against cytotoxicity of free FAs derived from membrane degradation, and remobilization of neutral lipids for membranes synthesis during bud dormancy release or pollen tube growth for example. Yet they also allow the storage of particular lipophilic compounds such as the antifungal oxylipins, isoprenoids in rubber particles and probably some vitamins that still need to be identified in plants. In addition, they probably have cryoprotectant properties in seeds and dormant buds, as well as in roots. Within cells, LDs are highly mobile organelles (reviewed in non-plant cells by Kilwein and Welte, 2019) and share multiple transient contact sites with other organelles such as the ER and peroxisomes of course, but also mitochondria, vacuole, plasma membrane (reviewed in Scholz et al., 2022) and chloroplast (Brocard et al., 2017). The nature of such LD-organelle contacts is not well defined in plant. A continuity between the LD monolayer and the cytosolic leaflet of the ER membrane is described for example in the case of LD-ER connections during LD nucleation and growth. Membrane contact sites (MCS), which are defined as the close (less than 30 nm) apposition of two membranes without any fusion event, mediated by tethering proteins, have recently been proposed to occur between LDs and plasma membrane, through the tethering of SEED LD PROTEIN (SLDP) and LD-PLASMA MEMBRANE ADAPTOR (LIPA) proteins (Krawczyk et al., 2022b). However, the confocal microscopic data provided in this study do not allow to firmly exclude any membrane fusion event and thus to firmly identify *sensu stricto* MCS at the site of LD-plasma membrane junctions. Similarly, the exact identity of the connections described between LDs and peroxisomes (Reviewed in Esnay et al., 2020) still remains to be determined. Whatever their exact nature and structure, these contacts would favor the necessary exchange of lipids (reviewed in Michaud and Jouhet, 2019; Scholz et al., 2022) occurring under stress condition, for example for membrane remodeling (cf. **Figure 2**). In addition, such contacts would also allow the transfer of enzymes and other hydrophobic proteins that would more easily transit within the cell by riding piggyback on LDs than through the aqueous cytosol. Given the high number of proteins identified in LD proteomes, different additional functions of plant LDs most probably remain to be discovered. Notably, involvement in resistance to ER stress or to mitochondrial damage during autophagy, that have been described in animal cells, are also presumably supported by LDs in plant cells.

Nowadays, LDs are the target of choice for researchers and industrialists to modify the lipid metabolism of plants for biotechnological purposes (*e.g.* increase of oil content, production of high-value proteins requiring hydrophobic environment). However, the involvement of LDs in so many physiological functions of the plant should be taken into account during such studies in order to minimize the impact on the development (and thus the yield) of the plant.

## Author contributions

Each author participated to the writing of this review. All authors contributed to the article and approved the submitted version.
